# The causal relationship between smoking, alcohol consumption, and sepsis: A 2-sample mendelian randomized study

**DOI:** 10.1097/MD.0000000000042102

**Published:** 2025-04-11

**Authors:** Yonghan Luo, Xiaotao Yang, Yanchun Wang

**Affiliations:** a Second Department of Infectious Disease, Kunming Children’s Hospital, Kunming, Yunnan, China; b Faculty of Life Science and Technology, Kunming University of Science and Technology, Kunming, Yunnan, China.

**Keywords:** alcohol consumption, Mendelian randomization, sepsis, smoking

## Abstract

A Mendelian randomization (MR) study was used to explore whether there is a causal relationship between smoking, alcohol consumption, and sepsis. Genome-wide association studies data on sepsis, alcohol consumption, and 3 smoking behaviors including smoking initiation, age of initiation, and cigarettes per day were extracted from public databases. The inverse variance weighting (IVW), weighted median, and MR-Egger regression method were used to analyze the causal association between smoking, alcohol consumption, and sepsis. Forest plots of the causal relationship between smoking, alcohol consumption, and sepsis were plotted. The MR Analysis based mainly on IVW showed a causal relationship between cigarettes per day and sepsis (OR = 1.24, 95% CI = 1.11–1.37, *P* = .000). Heterogeneity and horizontal pleiotropy were excluded by sensitivity analysis. MR Analysis showed that there was no causal association between sepsis and smoking initiation, age of initiation, and alcohol consumption. There is a positive causal effect between cigarettes per day and the risk of sepsis. However, there was insufficient evidence for a causal relationship between sepsis and smoking initiation, age of initiation, and alcohol consumption.

## 1. Introduction

Sepsis is defined as an immune dysregulation due to infection and can cause life-threatening organ function impairment.^[1]^ Although the incidence of sepsis has decreased by 37.0% and the case fatality rate by 52.8% from 1990 to 2017, there were an estimated 48.9 million new cases of sepsis and 11.0% sepsis-related deaths in 2017 alone, accounting for 19.7% of all deaths globally.^[2]^ Sepsis is a major factor in the global burden of disease, with the majority of sepsis cases and deaths occurring in low- and middle-income countries.^[3]^

Certain lifestyle habits may affect the incidence of sepsis, and the role of smoking in increasing the susceptibility of sepsis has been paid attention by researchers.^[4–6]^ A prospective case-control study demonstrated smoking as a risk factor for sepsis hospitalizations.^[7]^ A meta-analysis of 5 studies suggested that compared with nonsmokers, smokers with sepsis had a significantly higher risk of death (HR = 1.62).^[8]^ In addition, another lifestyle of chronic alcohol investigation is also thought to be associated with a high incidence of sepsis.^[9,10]^ However, these studies to explore the relationship between alcohol consumption, smoking and sepsis are usually retrospective, lack of high-quality randomized controlled trials. It is unknown whether there is a causal relationship between smoking, alcohol consumption and sepsis.

Mendelian randomization (MR) is a genetic epidemiological method that uses single nucleotide polymorphisms (SNP) as instrumental variable to investigate the causal effects of exposure on disease outcome. In recent years, MR has been widely used in the world to evaluate the potential causal relationship between various diseases and risk factors. In the case of sepsis, a 2-sample randomized study has been used to analyze the relationship between body mass index and sepsis,^[11]^ which provides new ideas for our research. Considering the above factors, we used a MR study to explore whether there is a causal relationship between smoking, alcohol consumption, and sepsis. It is hoped to provide evidence-based basis for the prevention and treatment of sepsis.

## 2. Methods

### 2.1. Study design

**Figure 1.**
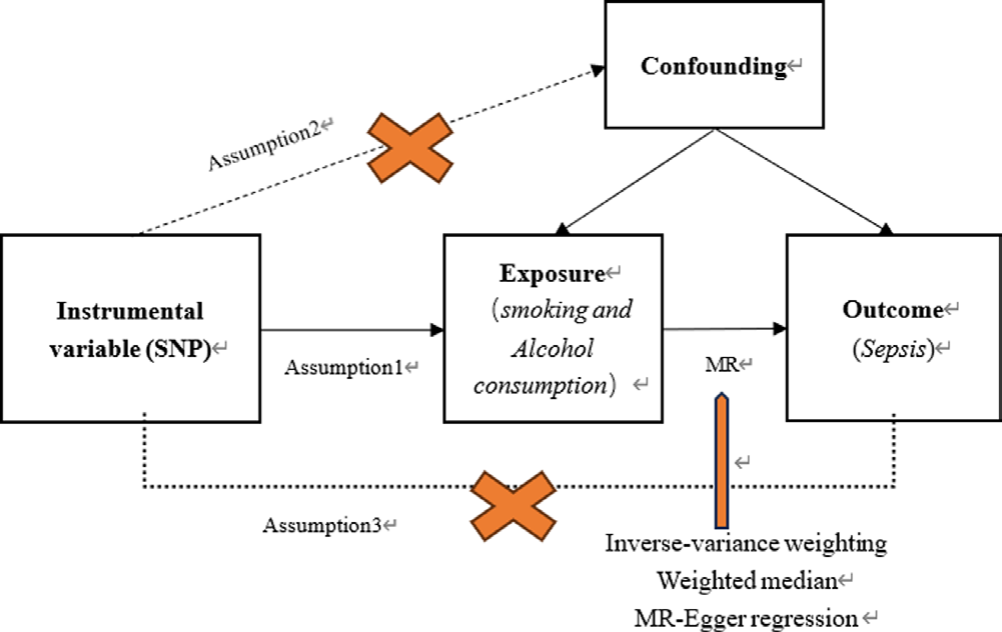
Flow chart of 2-sample MR. Assumption 1: the genetic variants proposed as IV should be robustly associated with the exposure. Assumption 2: IV should not be associated with potential confounders. Assumption 3: IV should affect the risk of the outcome merely through the risk factor, not via alternative pathways. IV = instrumental variables, MR = Mendelian randomization.

In this study, a 2 sample MR were used to analyze the causal relationship between smoking, alcohol consumption and sepsis. Firstly, SNP from open databases significantly related to smoking and alcohol consumption were selected as instrumental variables (IV). Secondly, the inverse variance weighting (IVW), weighted median, and MR-Egger regression method were used to analyze the causal association between smoking, alcohol consumption, and sepsis. Finally, sensitivity analysis was performed on the results. This study follows 3 key assumptions of the MR method (Fig. 1): there is a robust association between IVs and exposures; IVs should not be associated with any confounding factors; IVs can merely affect the risk of sepsis by influencing smoking and drinking behaviors.

### 2.2. Data sources

The SNPs of outcome variable (11,643 sepsis cases and 486,484 control cases; 12,243,539 SNPs) in this study came from the genome-wide association studies (GWAS) data in IEU OpenGwas source database (https://gwas.mrcieu.ac.uk). In terms of exposure variables, a complete dataset was obtained from the GWAS and sequencing consortium of alcohol and nicotine use (https://genome.psych.umn.edu/index.php/GSCAN) database. They conducted a meta-analysis of over 30 GWAS in over 1.2 million participants with European ancestry on nicotine and substance use. Then alcohol consumption, and 3 smoking behaviors including smoking initiation, age of initiation, and cigarettes per day were extracted from the databases.^[12]^ By defining the *P*-value and linkage disequilibrium conditions (*P* < 5 × 10^−8^, *R*^2^ < 0.001, kb = 10,000), the selected SNPs were used as IVs to replace risk exposure factors. The intensity of the correlation between the exposure factors and SNPs was judged by the *F* value (*F* = (β/SE)^2^). When *F* > 10, it was generally considered that there was no bias of IVs, and the SNPs with *F* ≤ 10 were screened out. Detailed SNPs data for each exposure phenotype are provided in supplementary tables (Tables S1 to S4, Supplemental Digital Content, http://links.lww.com/MD/O666).

### 2.3. Statistical analysis

The “TwoSampleMR” package in R software version 4.2.2 was performed for statistical analysis. IVW method was used to analyze the relationship of IVs between exposures and sepsis. When there is weak instrumental bias and no horizontal pleiotropic balance, the IVW method was known to produce reliable estimates.^[13]^ The weighted median and MR-Egger method were used as supplementary methods. The weighted median is reliable when up to 50% of the SNPs are invalid as an instrumental variable.^[14]^ The weighted linear regression of effect estimates by MR–Egger regression can provide a valid assessment of causal effect even when all SNPs are invalid instruments.^[15]^ Sensitivity analysis: heterogeneity was tested using Cochran *Q* test. The Egger-intercept test was used to test horizontal pleiotropy. In this study, the leave-one-out method was also used to analyze the sensitivity of the results. The genetic relationships between exposures and the risk of sepsis were displayed visually using scatter plots and funnel plots. The results were expressed as odds ratio and 95% confidence interval. *P* < .05 was considered statistically significant.

## 3. Results

### 3.1. Selection of instrumental variable

Detailed information on each SNP obtained by screening each exposure phenotype with P values and linkage disequilibrium is available in supplementary table (Tables S1 to S4, Supplemental Digital Content, http://links.lww.com/MD/O666). The *F*-values in the study were all >10, indicating that the results of this study were reliable without bias.

### 3.2. The total causal effect between smoking or drinking behavior and sepsis

**Figure 2.**
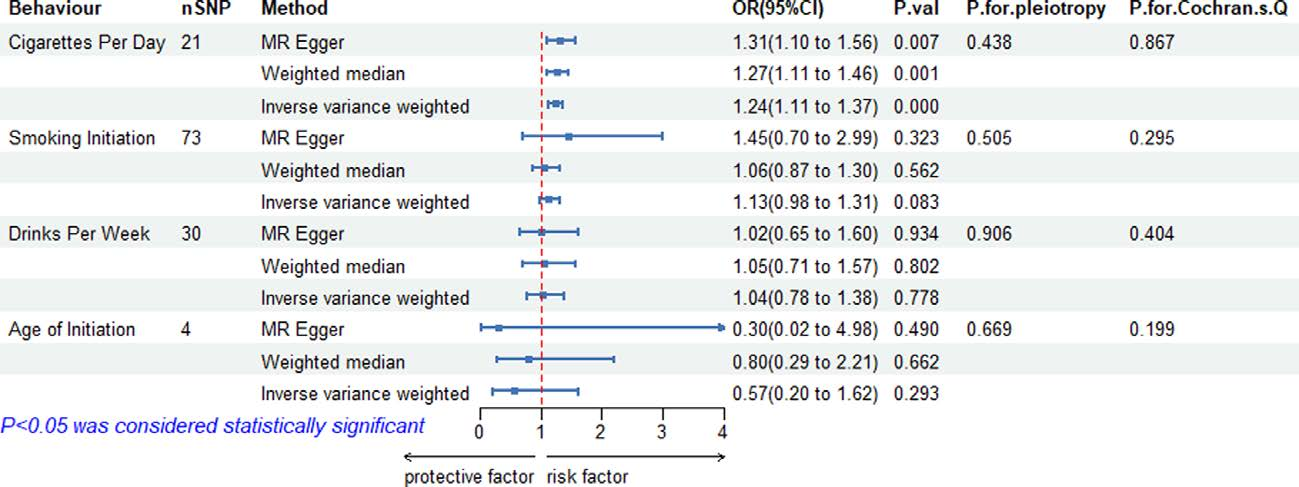
Forest plot of causal relationship between smoking, alcohol consumption, and sepsis.

The MR analysis based on IVW showed a positive causal relationship between cigarettes per day and sepsis (OR = 1.24, 95% CI = 1.11–1.37, *P* = .000). Moreover, weighted median (OR = 1.27, 95% CI = 1.11–1.46, *P* = .001) and MR–Egger method (OR = 1.31, 95% CI = 1.10–1.56, *P* = .007) were consistent with IVW, showing a positive causal relationship between cigarettes per day and sepsis. However, The MR analysis showed no causal relationship between smoking initiation (OR = 1.13, 95% CI = 0.98–1.31, *P* = .083), drinks per week (OR = 1.04, 95% CI = 0.78–1.38, *P* = .778), age of initiation (OR = 0.57, 95% CI = 0.20–163, *P* = .293), and sepsis (see Fig. 2).

### 3.3. Sensitivity analysis

**Figure 3.**
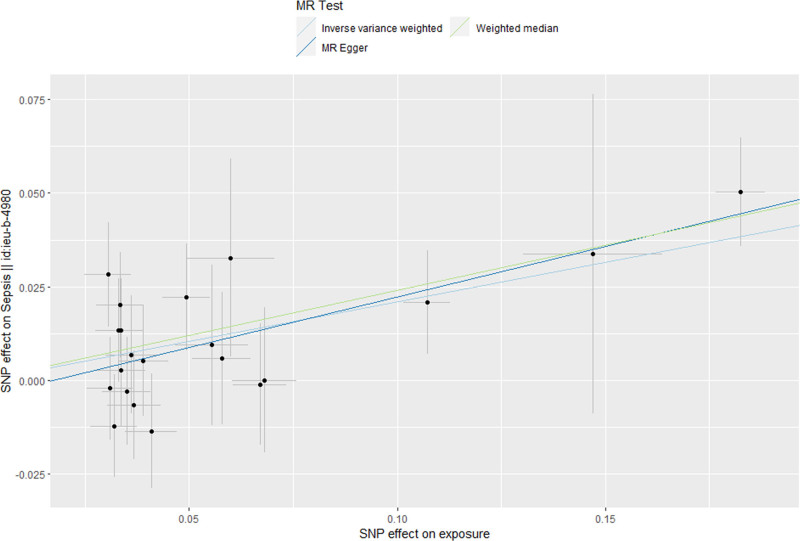
Funnel plot of SNPs associated with cigarettes per day and their risk of sepsis. SNPs = single nucleotide polymorphisms.

**Figure 4.**
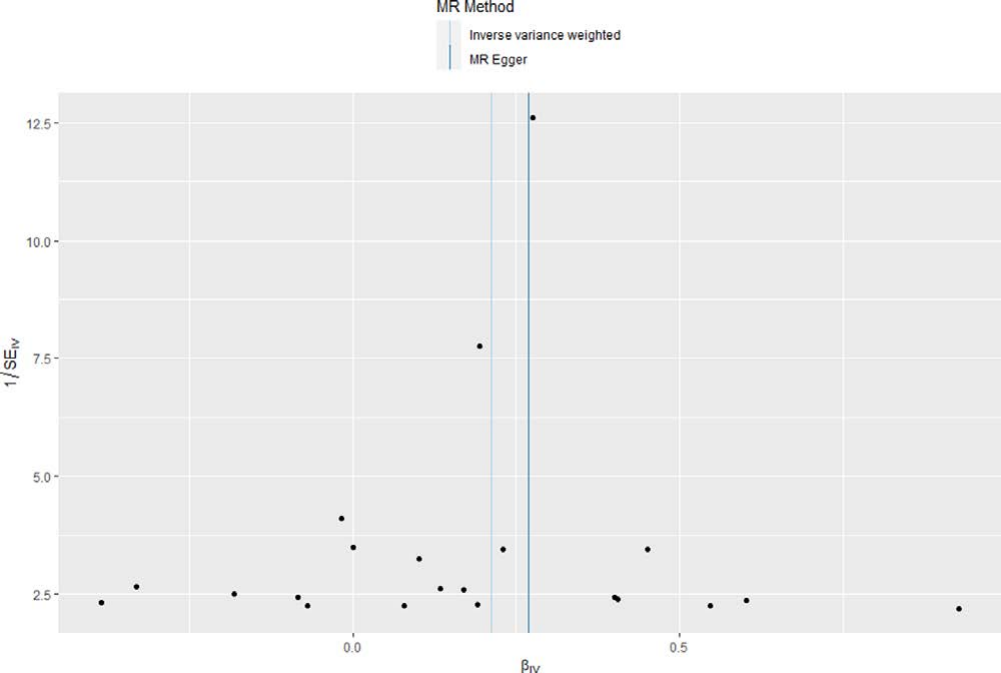
Scatter plot of SNPs associated with cigarettes per day and their risk of sepsis. SNPs = single nucleotide polymorphisms.

**Figure 5.**
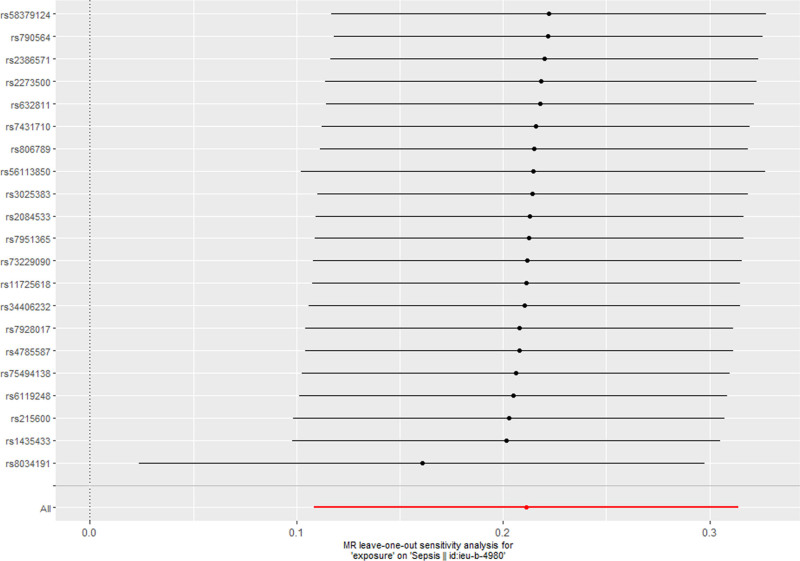
The leave-one-out analysis for cigarettes per day on sepsis.

Cochran *Q* test showed no heterogeneity among the SNP for cigarettes per day (*P* = .867) (Fig. 3). The result of MR-Egger regression intercept showed that there was no horizontal pleiotropy of correlation between cigarettes per day and sepsis (*P* = .438) (Fig. 4). The results of leave-one analysis revealed that there were no SNP of cigarettes per day that significantly affected the estimated effects of sepsis, suggesting a reliable causal relationship (Fig. 5). The results of sensitivity analyses for smoking initiation, drinks per week, and age of initiation can be seen in Fig. 2, showing no significant horizontal pleiotropy and heterogeneity.

## 4. Discussion

In the study, we used a MR study to explore whether there is a causal relationship between smoking, alcohol consumption, and sepsis. MR displayed a positive causal effect between cigarettes per day and the risk of sepsis. However, there was insufficient evidence for a causal relationship between sepsis and smoking initiation, age of initiation, and alcohol consumption. It is hoped to provide evidence-based basis for the prevention and treatment of sepsis.

At present, some studies^[4–6]^ suggest that some lifestyles are related to the susceptibility and prognosis of sepsis, but there is a lack of large sample randomized controlled studies on whether there is a causal relationship between smoking, alcohol consumption and sepsis. In terms of smoking alone, some researchers^[8,16,17]^ believe that there is a positive causal relationship between smoking and sepsis and smokers have a worse prognosis of sepsis. McMillan et al^[7]^ believe that there is a significant positive correlation between smoking and sepsis hospitalization and advocate smoking cessation to reduce the prevalence of sepsis. However, 1 study^[18]^ suggests that nicotine in tobacco may have a protective effect against sepsis-related inflammatory damage, which appears to involve inhibition of neutrophil activity in the tissues. Therefore, whether smoking has a promoting or protective causal effect on sepsis is currently controversial. Our MR study investigated the causal relationship between 3 smoking behaviors including smoking initiation, age of initiation, and cigarettes per day and sepsis. Our results showed a positive causal relationship between cigarettes per day and sepsis, but there was insufficient evidence to conclude that smoking initiation and age of initiation were causal for sepsis. It showed that the amount of tobacco has a direct impact on the occurrence of sepsis. Because both the variables of smoking initiation and age of initiation can only indicate the experience of smoking, but not the amount of tobacco consumed. In contrast, many smokers quit at a certain age, and their risk for sepsis decreases. Cigarettes per day, however, means a large amount of tobacco intake over the years, which greatly increases the risk of sepsis. Our findings will be beneficial to the selection of high-risk groups for sepsis and suggest that reducing tobacco smoking may reduce the risk of sepsis, which will be a warning to a large number of smokers around the world.

The mechanisms by which smoking increases the susceptibility to sepsis remain unclear. One study^[19]^ suggested that smoking increased the risk of sepsis by increasing the incidence of cardiovascular and cerebrovascular diseases. It means that smoking was indirectly through the confounding factor of cardiovascular and cerebrovascular diseases rather than directly increased the risk of sepsis. However, it does not meet assumption 2 (instrumental variables should not be associated with potential confounders) that MR must follow. Smoking contains more than 7000 different chemicals. The main harmful components include nicotine, acrolein, aromatic hydrocarbons, heavy metals, and nitrogen (ROS and RNS), which can damage cellular and subcellular targets such as lipids, proteins, and nucleic acids, and play a key role in cigarette-induced inflammation.^[20,21]^ There is also evidence that tobacco components have a significant effect on immune regulation, and immune disorders promote inflammation.^[20]^

It is well known that alcohol consumption is also a risk factor for many inflammatory diseases.^[22,23]^ In terms of the relationship between alcohol consumption and sepsis, some studies^[24–26]^ suggest that chronic alcohol abuse contributes to increased susceptibility to sepsis. On the contrary, our study examined the association between weekly alcohol consumption and sepsis, finding no significant causal relationship. However, the sample we included was only weekly drinkers rather than alcohol abusers. In the future, we should look for SNPs in daily drinkers to further answer the causal relationship between alcohol and sepsis.

Our study has some strengths. Firstly, the sample of this study is large, which can largely avoid the influence of confounding factors on the results. Secondly, MR studies can reliably estimate the causal effect between risk factors and diseases, avoiding the reverse causality caused by traditional observational studies. However, this study has some limitations. First, the data used in this study were from European populations, so the conclusion cannot be generalized to all populations. In the future, larger GWAS in other continents are needed to verify the results. Second, more detailed cohort data such as age and gender were not available for further subgroup analysis.

## 5. Conclusion

There is a positive causal effect between cigarettes per day and the risk of sepsis. However, there was insufficient evidence for a causal relationship between sepsis and smoking initiation, age of initiation, and alcohol consumption.

## Author contributions

**Conceptualization:** Yonghan Luo, Yanchun Wang.

**Data curation:** Yonghan Luo, Yanchun Wang, Xiaotao Yang.

**Formal analysis:** Yonghan Luo, Xiaotao Yang.

**Funding acquisition:** Yanchun Wang.

**Investigation:** Yonghan Luo, Xiaotao Yang.

**Methodology:** Yonghan Luo, Yanchun Wang.

**Project administration:** Yanchun Wang.

**Resources:** Yanchun Wang.

**Software:** Yonghan Luo.

**Supervision:** Yanchun Wang.

**Writing – original draft:** Yonghan Luo, Yanchun Wang.

**Writing – review & editing:** Yonghan Luo, Yanchun Wang.

## Supplementary Material



## References

[1] Singer M Deutschman CS Seymour CW. The third international consensus definitions for sepsis and septic shock (sepsis-3). JAMA. 2016;315:801–10. 26903338 10.1001/jama.2016.0287 PMC4968574

[2] Rudd KE Johnson SC Agesa KM. Global, regional, and national sepsis incidence and mortality, 1990-2017: analysis for the Global Burden of Disease Study. Lancet. 2020;395:200–11. 31954465 10.1016/S0140-6736(19)32989-7 PMC6970225

[3] Rudd KE Kissoon N Limmathurotsakul D. The global burden of sepsis: barriers and potential solutions. Crit Care. 2018;22:232. 30243300 10.1186/s13054-018-2157-z PMC6151187

[4] Ni J Lin Z Wu Q. Discharge against medical advice after hospitalization for sepsis: predictors, 30-day readmissions, and outcomes. J Emerg Med. 2023;65:e383–92. 37741736 10.1016/j.jemermed.2023.05.014

[5] Stensrud VH Gustad LT Damås JK Solligård E Krokstad S Nilsen TIL. Direct and indirect effects of socioeconomic status on sepsis risk and mortality: a mediation analysis of the HUNT Study. J Epidemiol Community Health. 2023;77:168–74. 36707239 10.1136/jech-2022-219825

[6] Hassan N Slight R Weiand D. Preventing sepsis; how can artificial intelligence inform the clinical decision-making process? A systematic review. Int J Med Inform. 2021;150:104457. 33878596 10.1016/j.ijmedinf.2021.104457

[7] McMillan M Davis JS. Acute hospital admission for sepsis: an important but under-utilised opportunity for smoking cessation interventions. Aust N Z J Public Health. 2010;34:432–3. 20649788 10.1111/j.1753-6405.2009.00580.x

[8] Zhang N Liu Y Yang C. Association between smoking and risk of death in patients with sepsis: A systematic review and meta-analysis. Tob Induc Dis. 2022;20:65. 35903643 10.18332/tid/150340 PMC9284521

[9] Moss M. Epidemiology of sepsis: race, sex, and chronic alcohol abuse. Clin Infect Dis. 2005;41(Suppl 7):S490–7. 16237652 10.1086/432003

[10] Margoles LM Mittal R Klingensmith NJ. Chronic alcohol ingestion delays t cell activation and effector function in sepsis. PLoS One. 2016;11:e0165886. 27861506 10.1371/journal.pone.0165886 PMC5115670

[11] Wang J Hu Y Zeng J. Exploring the causality between Body Mass Index and sepsis: a two-sample mendelian randomization study. Int J Public Health. 2023;68:1605548. 37205044 10.3389/ijph.2023.1605548 PMC10186272

[12] Liu M Jiang Y Wedow R; 23andMe Research Team. Association studies of up to 1.2 million individuals yield new insights into the genetic etiology of tobacco and alcohol use. Nat Genet. 2019;51:237–44. 30643251 10.1038/s41588-018-0307-5 PMC6358542

[13] Hemani G Bowden J Davey Smith G. Evaluating the potential role of pleiotropy in Mendelian randomization studies. Hum Mol Genet. 2018;27:R195–208. 29771313 10.1093/hmg/ddy163 PMC6061876

[14] Bowden J Davey Smith G Haycock PC Burgess S. Consistent estimation in mendelian randomization with some invalid instruments using a weighted median estimator. Genet Epidemiol. 2016;40:304–14. 27061298 10.1002/gepi.21965 PMC4849733

[15] Burgess S Thompson SG. Interpreting findings from Mendelian randomization using the MR-Egger method. Eur J Epidemiol. 2017;32:377–89. 28527048 10.1007/s10654-017-0255-x PMC5506233

[16] Alroumi F Abdul Azim A Kergo R Lei Y Dargin J. The impact of smoking on patient outcomes in severe sepsis and septic shock. J Intensive Care. 2018;6:42. 30065844 10.1186/s40560-018-0312-x PMC6064183

[17] Szakmany T Lundin RM Sharif B; Welsh Digital Data Collection Platform Collaborators. Sepsis Prevalence and outcome on the general wards and emergency departments in wales: results of a multi-centre, observational, point prevalence study. PLoS One. 2016;11:e0167230. 27907062 10.1371/journal.pone.0167230 PMC5132245

[18] Özdemir-Kumral ZN Özbeyli D Özdemir AF. Protective effect of nicotine on sepsis-induced oxidative multiorgan damage: role of neutrophils. Nicotine Tob Res. 2017;19:859–64. 27613897 10.1093/ntr/ntw198

[19] López-Mestanza C Andaluz-Ojeda D Gómez-López JR Bermejo-Martín JF. Clinical factors influencing mortality risk in hospital-acquired sepsis. J Hosp Infect. 2018;98:194–201. 28882641 10.1016/j.jhin.2017.08.022

[20] Liu Y Lu L Yang H. Dysregulation of immunity by cigarette smoking promotes inflammation and cancer: a review. Environ Pollut. 2023;339:122730. 37838314 10.1016/j.envpol.2023.122730

[21] Caliri AW Tommasi S Besaratinia A. Relationships among smoking, oxidative stress, inflammation, macromolecular damage, and cancer. Mutat Res Rev Mutat Res. 2021;787:108365. 34083039 10.1016/j.mrrev.2021.108365 PMC8287787

[22] Forsyth CB Voigt RM Swanson GR. Alcohol use disorder as a potential risk factor for COVID-19 severity: a narrative review. Alcohol Clin Exp Res. 2022;46:1930–43. 36394508 10.1111/acer.14936 PMC9722573

[23] Morojele NK Shenoi SV Shuper PA Braithwaite RS Rehm J. Alcohol use and the risk of communicable diseases. Nutrients. 2021;13:3317. 34684318 10.3390/nu13103317 PMC8540096

[24] Jeansonne D Jeyaseelan S. Role of an anti-aging molecule in a toxic lifestyle: relevance for alcohol effects on sepsis. Alcohol Clin Exp Res. 2021;45:912–5. 33650706 10.1111/acer.14587

[25] Jin L Batra S Jeyaseelan S. Diminished neutrophil extracellular trap (NET) formation is a novel innate immune deficiency induced by acute ethanol exposure in polymicrobial sepsis, which can be rescued by CXCL1. PLoS Pathog. 2017;13:e1006637. 28922428 10.1371/journal.ppat.1006637 PMC5626520

[26] Klingensmith NJ Fay KT Lyons JD. Chronic alcohol ingestion worsens survival and alters gut epithelial apoptosis and CD8+ T cell function after pseudomonas aeruginosa pneumonia-induced Sepsis. Shock. 2019;51:453–63. 29664837 10.1097/SHK.0000000000001163 PMC6191382

