# Supplementary material for: The causal relationship between smoking, alcohol consumption, and sepsis: A 2-sample mendelian randomized study
Source: Medicine (Baltimore). 2025 Apr 11;104(15):e42102. doi: 10.1097/MD.0000000000042102 (PMC11999439; doi:10.1097/MD.0000000000042102)
Supplement: Supplementary file 1 [file medi-104-e42102-s001.pdf]

Supplementary Table 1. SNPs associated with Age of Initiation.

SNP	Other allele	Effect allele	beta	SE	pval	F
rs7599208	C	T	-0.019617115	0.002779844	1.70243E-12	49.80000961
rs3768886	G	C	0.017140245	0.002965813	7.50238E-09	33.40000423
rs11915747	C	G	0.020989626	0.002891342	3.88595E-13	52.69998247
rs624833	T	G	0.017294481	0.003006032	8.75392E-09	33.09999043
rs11780471	G	A	0.037850385	0.005805984	7.06822E-11	42.50000032
rs140485736	G	A	0.065453218	0.011534614	1.39091E-08	32.20000029
rs319748	G	A	-0.017033929	0.003074296	3.01166E-08	30.69999505

Supplementary Table 2. 93 SNPs associated with Smoking Initiation.

SNP	Other allele	Effect allele	beta	SE	pval	F
rs301805	T	G	0.021467915	0.003613287	2.82631E-09	35.30000908
rs3001723	G	A	0.033511798	0.003898302	8.21759E-18	73.90001009
rs6669839	C	T	0.026004122	0.004395499	3.29706E-09	34.99999793
rs1937443	C	G	0.026064233	0.003586972	3.69301E-13	52.80000036
rs7555507	C	T	-0.02414444	0.00355604	1.12368E-11	46.10000781
rs11162019	C	T	-0.020509083	0.003701494	3.01165E-08	30.70000073
rs12042107	T	C	-0.022283376	0.003568196	4.23806E-10	38.99999693
rs12027999	T	C	-0.0330855	0.00533915	5.76326E-10	38.39999431
rs2046850	C	T	-0.024813853	0.004478422	3.01165E-08	30.70000039
rs6728726	T	C	0.035448593	0.004732794	6.88774E-14	56.0999922
rs1004787	G	A	0.02992312	0.003571399	5.35869E-17	70.19998237
rs1518393	A	C	0.020535744	0.003658939	1.9944E-08	31.49999937
rs7585579	C	G	0.022399646	0.0037281	1.87447E-09	36.10000014
rs2672851	C	G	-0.030505242	0.003737983	3.32584E-16	66.60001169
rs35702515	G	T	0.025244173	0.004230933	2.42288E-09	35.60000013
rs13030994	G	A	0.036092466	0.003556296	3.35145E-24	103.0000201
rs1445649	T	C	0.023993234	0.003564837	1.69049E-11	45.30000246
rs12474587	G	T	0.027632862	0.003582345	1.22295E-14	59.49999363
rs6433897	T	C	0.022448266	0.004058094	3.17094E-08	30.59999775
rs2107300	C	G	-0.027201037	0.004925332	3.33866E-08	30.50000441

rs4674993	A	G	-0.02521221	0.004436185	1.32113E-08	32.30000084
rs11721059	C	T	0.019935687	0.003563355	2.21081E-08	31.29999228
rs12632110	A	G	-0.023376824	0.003752923	4.69528E-10	38.79999828
rs11712680	A	C	-0.027047624	0.004578428	3.4708E-09	34.90000291
rs6788098	A	T	-0.031346052	0.003689048	1.94458E-17	72.19998166
rs7631735	G	T	0.020227305	0.003644707	2.86037E-08	30.80000623
rs1154693	A	G	0.032621668	0.004912323	3.12022E-11	44.09999056
rs292071	T	C	0.023582199	0.004009094	4.04906E-09	34.60000181
rs993700	T	C	-0.025927991	0.004291632	1.52667E-09	36.4999933
rs1160685	C	G	0.020772436	0.00358893	7.12638E-09	33.50000591
rs13145728	G	C	-0.023251231	0.003662633	2.17808E-10	40.30000441
rs10001365	G	A	-0.024991752	0.00364155	6.74559E-12	47.09999385
rs6893752	A	G	-0.024099534	0.004073565	3.29706E-09	34.99999552
rs4571506	C	T	-0.027574664	0.003568807	1.10477E-14	59.70000043
rs12186738	G	T	-0.033264435	0.005020511	3.4559E-11	43.89999358
rs72789632	C	T	-0.032885561	0.005286275	4.94208E-10	38.70000364
rs1385108	C	T	0.024661699	0.004156729	2.97525E-09	35.19999184
rs4044321	A	G	-0.027841695	0.003710577	6.22152E-14	56.3000067
rs222449	A	T	-0.025320823	0.004427963	1.07538E-08	32.69999365
rs10498846	C	T	0.020610294	0.003555613	6.76924E-09	33.60000972
rs2892512	T	C	0.027730657	0.003986002	3.4757E-12	48.39999242

rs3800227	A	G	0.022812119	0.004058094	1.8943E-08	31.59999774
rs240963	T	C	-0.041044352	0.004837123	2.15196E-17	72.00000808
rs4236259	T	G	-0.024768919	0.00355661	3.30291E-12	48.49998623
rs10250550	T	C	-0.020329029	0.003610663	1.79922E-08	31.70000711
rs13246563	C	G	-0.023463104	0.003737983	3.45296E-10	39.40000595
rs12112638	A	G	-0.024525968	0.004042986	1.3089E-09	36.80000178
rs11768481	C	A	-0.023201667	0.003763807	7.07443E-10	38.00000794
rs12333760	T	C	-0.029046684	0.004801269	1.45032E-09	36.59999638
rs10233018	A	G	0.027069038	0.003557407	2.75788E-14	57.89999589
rs10279261	G	A	-0.02141936	0.003662633	4.97288E-09	34.20000522
rs1565735	T	A	-0.037618034	0.004461299	3.39572E-17	71.09998797
rs7829715	T	C	-0.026894575	0.00355604	3.93669E-14	57.20000893
rs12545053	A	G	0.020280798	0.003636678	2.45071E-08	31.09999937
rs10956809	G	C	-0.020781065	0.003579751	6.43005E-09	33.70000485
rs1899896	C	T	0.026448125	0.003886909	1.01464E-11	46.30000431
rs4543592	T	C	0.021931432	0.003562439	7.44656E-10	37.89999093
rs10114490	G	A	-0.025514755	0.004531706	1.79922E-08	31.70000652
rs2378662	G	A	0.020948155	0.003566451	4.26252E-09	34.49999517
rs10905461	T	C	-0.023955389	0.004145052	7.50239E-09	33.40000169
rs10159545	C	G	0.026250115	0.003727266	1.88514E-12	49.59999644
rs7921378	G	C	-0.02546008	0.003558155	8.34187E-13	51.19999737

			4			
rs12356821	G	C	0.039370036	0.005049101	6.31782E-15	60.79998994
rs9423279	C	G	-0.020513169	0.003708276	3.17094E-08	30.59999921
rs4523689	A	G	-0.020609101	0.003643209	1.54173E-08	31.99999592
rs6265	C	T	-0.031786296	0.004578428	3.84888E-12	48.20000339
rs7929518	A	G	0.024237689	0.004284658	1.54172E-08	32.00000841
rs7938812	T	G	0.043791401	0.003636678	2.14765E-33	144.9999928
rs11057005	A	G	-0.02092978	0.003578917	4.9729E-09	34.19999743
rs4759228	G	C	-0.021691308	0.003934132	3.51528E-08	30.40000304
rs7969559	A	G	-0.024375606	0.003959459	7.44655E-10	37.89999411
rs1971318	C	T	0.028507422	0.004925332	7.12638E-09	33.50000479
rs3904512	G	A	-0.021158885	0.003576504	3.29704E-09	35.00000659
rs9540729	A	T	-0.019552223	0.003557891	3.89709E-08	30.20000439
rs7322872	C	T	-0.025571284	0.004334739	3.65375E-09	34.79999366
rs76214862	A	C	-0.024990316	0.004547454	3.89711E-08	30.19999467
rs1435741	G	A	0.029415124	0.003590951	2.58081E-16	67.09998281
rs12441907	C	A	-0.029205064	0.00452262	1.06409E-10	41.70000689
rs7197072	C	T	-0.024767214	0.004168596	2.82632E-09	35.29999943
rs12923427	C	T	-0.023908663	0.004372398	4.54913E-08	29.9000013
rs4785836	T	C	-0.020470447	0.003658939	2.2108E-08	31.2999983
rs1050847	C	T	-0.02162311	0.00358893	1.69163E-09	36.30000637

			9			
rs11658881	A	G	0.020135721	0.003610663	2.45071E-08	31.100005
rs11078713	A	G	-0.020172092	0.00360561	2.21079E-08	31.30000315
rs7224742	C	T	-0.020709882	0.00365531 8	1.46438E-08	32.09999159
rs72896886	G	C	-0.026888489	0.004837123	2.71671E-08	30.90000385
rs6508144	C	G	-0.020693519	0.003586016	7.89826E-09	33.3000016
rs11872397	G	A	-0.024772541	0.004094775	1.45032E-09	36.59999495
rs76608582	C	A	-0.049557467	0.008259578	1.97318E-09	35.99999855
rs1555445	A	T	0.022554797	0.003823396	3.65373E-09	34.80000308
rs56820925	C	T	-0.021863819	0.003877149	1.70893E-08	31.7999959
rs117143374	T	C	0.02928969	0.005269089	2.71672E-08	30.90000017
rs134529	T	C	-0.019983953	0.003660777	4.78992E-08	29.79999909

Supplementary Table 3. 23SNPs associated with Cigarettes Per Day.

SNP	Other allele	Effect allele	beta	SE	pval	F
rs2072659	C	G	−0.06525177	0.009246505	1.70E-12	49.80000378
rs2084533	C	T	0.033640552	0.005900943	1.19E-08	32.50000199
rs7431710	G	A	−0.034957881	0.005810196	1.78E-09	36.20000523
rs11725618	T	C	0.036064018	0.006157821	4.72E-09	34.29999854
rs1435433	G	A	0.030476587	0.005573535	4.55E-08	29.9000004
rs806789	C	A	−0.030854126	0.005532648	2.45E-08	31.1000002
rs215600	G	A	−0.049250294	0.005752503	1.11E-17	73.29999535
rs73229090	C	A	0.055489485	0.008762711	2.41E-10	40.10000355
rs58379124	T	C	0.06693935	0.006501724	7.37E-25	106.000014
rs790564	A	C	−0.040892693	0.00619302	4.03E-11	43.59999697
rs3025383	T	C	−0.057839414	0.00704521	2.22E-16	67.39999787
rs7951365	T	C	0.038951113	0.005967811	6.72E-11	42.60000229
rs75494138	C	T	0.059876284	0.010568232	1.46E-08	32.09999776
rs7928017	C	A	−0.03293136	0.005558481	3.13E-09	35.09999723
rs632811	A	G	−0.036713674	0.006410485	1.02E-08	32.80000056
rs8034191	T	C	0.182566723	0.005889249	5.39E-211	961.0000421

rs2386571	A	C	-0.031868709	0.005564518	1.02E-08	32.79999957
rs4785587	G	A	-0.033622317	0.00553496	1.24E-09	36.89999469
rs895330	C	G	-0.039004149	0.007016678	2.72E-08	30.90000287
rs34406232	C	A	-0.146992005	0.01669717	1.33E-18	77.49999773
rs56113850	T	C	0.107204606	0.005603675	1.39E-81	365.9999377
rs6119248	A	G	0.033383557	0.005618822	2.83E-09	35.29999704
rs2273500	T	C	0.068093833	0.007795528	2.44E-18	76.29999175

Supplementary Table 4. 37 SNPs associated with Drinks Per Week.

SNP	Other allele	Effect allele	beta	SE	pval	F
rs10753661	G	A	−0.011365553	0.002075057	4.32049E-08	29.99998802
rs28680958	G	A	−0.01358482	0.002368405	9.70235E-09	32.89999547
rs1260326	T	C	0.02381201	0.001984334	3.55292E-33	144.0000242
rs62135521	G	T	−0.026357553	0.004595229	9.70234E-09	32.89999704
rs528301	G	A	0.015591937	0.001948992	1.24419E-15	64.00000821
rs6739804	T	C	−0.01296884	0.002082022	4.6953E-10	38.799992
rs4233567	C	T	−0.012589937	0.002010854	3.82541E-10	39.19999712
rs28732378	A	G	−0.016727407	0.002190758	2.25046E-14	58.29998398
rs28712821	G	A	0.028333875	0.001974115	1.02473E-46	205.9999193
rs16854020	G	A	0.018083033	0.002906804	4.94207E-10	38.7000077
rs1229984	T	C	0.188115393	0.00617852	1.3257E-203	927.000044
rs78234152	G	A	0.027653801	0.00307075	2.14582E-19	81.09997971
rs13107325	C	T	−0.036454753	0.00391286	1.2007E-20	86.79999523
rs331939	G	A	−0.011901612	0.002029208	4.48723E-09	34.40000012
rs4916723	A	C	−0.011270904	0.001953155	7.89831E-09	33.29998838
rs55872	G	T	0.0127313	0.002268	1.99441E	31.49999

084			84	404	-08	073
rs10085696	A	G	-0.016051676	0.002494711	1.24058E-10	41.39999475
rs2299409	G	A	-0.010554626	0.001933458	4.78993E-08	29.79999648
rs6951574	T	C	0.012752539	0.001935761	4.46185E-11	43.40001195
rs28601761	C	G	0.011298868	0.001955067	7.50236E-09	33.40000867
rs55932213	A	G	0.012476712	0.002216004	1.79922E-08	31.69999698
rs2049045	G	C	-0.013768259	0.002505391	3.89708E-08	30.20000593
rs4752999	C	T	-0.01456432	0.002070082	1.9837E-12	49.50001458
rs4309187	A	C	0.014790756	0.002087558	1.38848E-12	50.19999156
rs17542254	A	G	0.013141799	0.002146047	9.14133E-10	37.49999267
rs1387766	G	A	-0.01082702	0.001983357	4.78995E-08	29.79998815
rs34704785	C	T	-0.010581507	0.001935138	4.54913E-08	29.89999838
rs1123285	C	G	-0.012418317	0.002049886	1.37779E-09	36.70000005
rs28929474	C	T	-0.047680929	0.007139656	2.41692E-11	44.59999832
rs153106	T	C	-0.013611756	0.001958577	3.65755E-12	48.29998663
rs79616692	G	C	0.018808515	0.003152314	2.42287E-09	35.60000624
rs11860773	T	C	-0.015005068	0.002443809	8.25043E-10	37.70000571
rs13332432	C	G	0.014004968	0.002140722	6.06324E-11	42.79999291
rs34121753	A	G	0.011069397	0.001950725	1.39091E-08	32.19999672
rs76640	G	A	-	0.002389	1.46867E	77.29999

332			0.021008064	441	-18	055
rs838145	G	A	-0.015797822	0.001940173	3.87259E-16	66.2999986
rs6106989	G	A	0.010899442	0.001983357	3.89712E-08	30.19998608
